# Von Hippel-Lindau disease: A case report

**DOI:** 10.1016/j.ijscr.2022.107417

**Published:** 2022-07-16

**Authors:** Durga Neupane, Alok Dahal, Nimesh Lageju, Lokesh Shekher Jaiswal, Ashim Kandel, Srista Manandhar

**Affiliations:** a Department of Surgery, B.P. Koirala Institute of Health Sciences, Dharan, Nepal; b Department of Surgery (Division of Neurosurgery), B.P. Koirala Institute of Health Sciences, Dharan, Nepal; c Department of Surgery (Division of CTVS), B.P. Koirala Institute of Health Sciences, Dharan, Nepal

**Keywords:** Case report, Von Hippel-Lindau disease, Hemangioblastoma, MRI, Resection, CNS

## Abstract

**Introduction and importance:**

Von Hippel-Lindau (VHL) disease is a rare autosomal dominantly inherited genetic condition. Von Hippel characterized the illness independently in 1911, and Lindau in 1926. Its prevalence is estimated to be about 1 in every 36,000 live births. VHL is characterized by the production of several benign and malignant tumors, as well as cysts in other organs. For proper prognosis, good clinical judgement and timely diagnosis is warranted.

**Case presentation:**

Herein, we report a case of a 50-year-old man with several central nervous system (CNS) lesions, retinal lesions, and renal cortical cysts with a diagnosis of VHL disease who was surgically treated. At a 3-month follow-up, he improved drastically with a marked alleviation of his signs and symptoms.

**Discussion:**

VHL is characterized by the creation of various benign and malignant tumors, as well as cysts in multiple organs, and is passed down through generations in an autosomal dominant pattern with near-complete penetrance. CNS lesions are surgically treated. Regular follow-up should be ensured.

**Conclusions:**

VHL disease is an extremely complicated disease with the need for diagnosis and genetic tests in the patient and family members, as well as intensive supervision of carriers of the mutated gene, thereby improving early diagnosis and successful treatment of the malignancies. The high cost of diagnostics and surgical therapies is a severe issue. Government care and financial assistance are critical considerations.

## Highlights

• Von Hippel-Lindau (VHL) disease is a rare autosomal dominantly inherited genetic condition.
• It is linked to a mutation in both alleles of the VHL gene, which is situated on the short arm of chromosome 3.
• VHL is characterized by the production of several benign and malignant tumors, as well as cysts in other organs.
• Affected people are more likely to develop retinal and central nervous system (CNS) hemangioblastomas.
• Definitive diagnosis with proper genetic counselling and treatment is recommended.

## 1 Introduction

Von Hippel-Lindau (VHL) disease is a rare autosomal dominantly inherited genetic condition [1]. Von Hippel characterized the illness independently in 1911 [2], and Lindau in 1926 [3]. Its prevalence is estimated to be about 1 in every 36,000 live births [4]. It is linked to a mutation in both alleles of the VHL gene, which is situated on the short arm of chromosome 3 [5]. VHL is characterized by the production of several benign and malignant tumors, as well as cysts in other organs. Affected people are more likely to develop retinal and central nervous system (CNS) hemangioblastomas, clear cell renal cell carcinomas (RCC), pheochromocytomas, pancreatic neuroendocrine tumors, and endolymphatic sac tumors (ELSTs) [6]. It imposes a significant impact on the patient and the family members. Multitude of signs and symptoms along with increased health care resources utilization has a global impact.

Herein, we report a case of a 50-year-old man with several CNS lesions, retinal lesions, and renal cortical cysts with a diagnosis of VHL disease who was surgically treated. This case report is reported according to SCARE guideline [7].

## 2 Case report

**Fig. 1:**
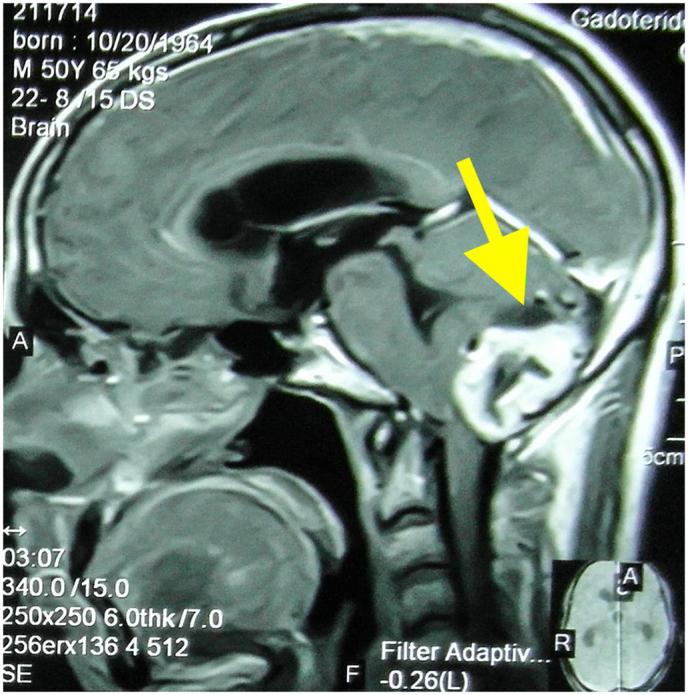
T1-weighted postcontrast MRI showing cerebellar hemangioblastoma (arrow).

**Fig. 2:**
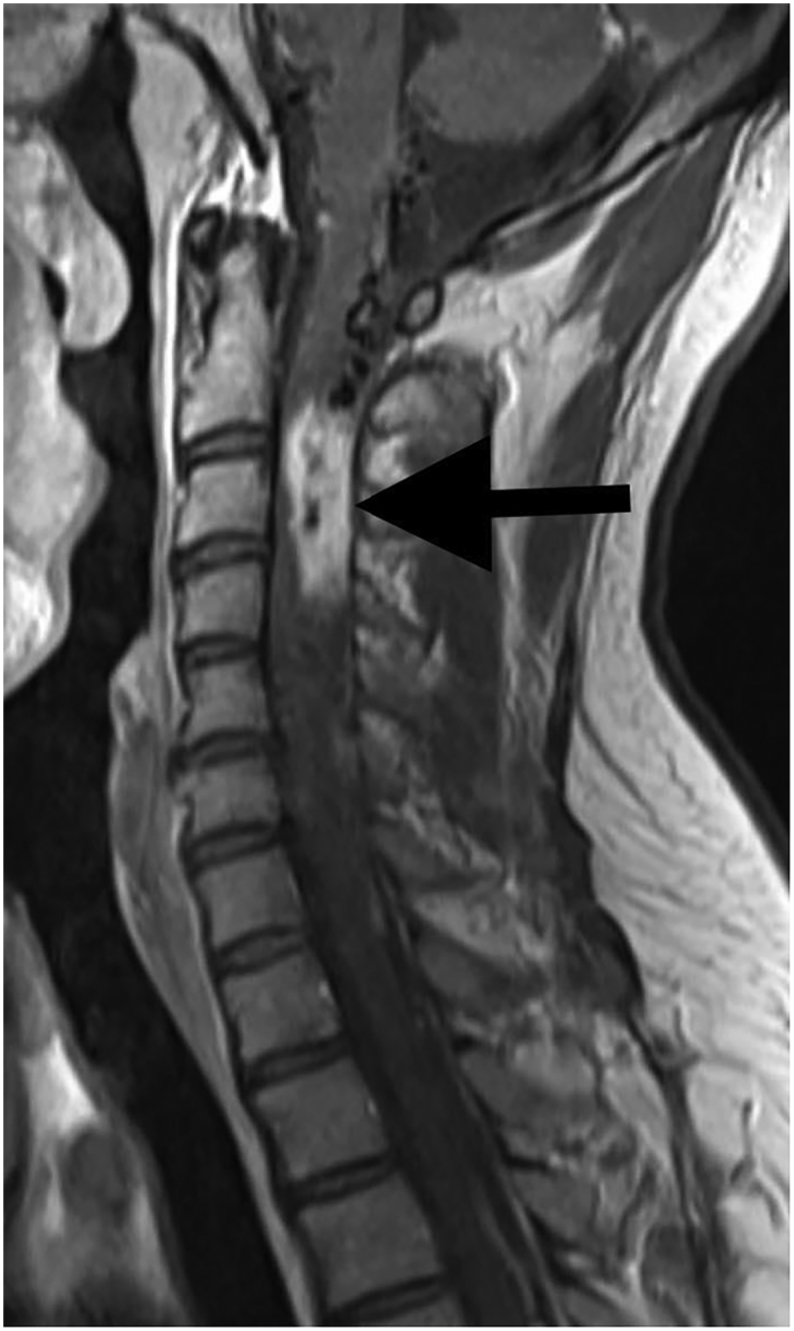
T1-weighted postcontrast MRI showing spinal hemangioblastoma at C2/C3/C4 cervical vertebrae level (arrow).

**Fig. 3:**
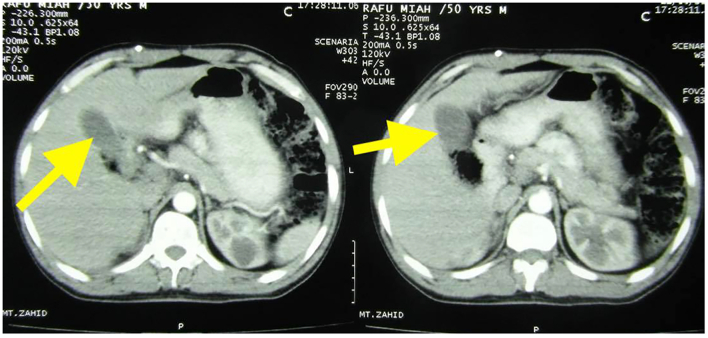
CECT abdomen showing space-occupying lesion in segment VI of the liver which on biopsy confirmed capillary liver hemangioblastoma (arrow).

A 50-year-old gentleman presented to the Emergency department with complains of abnormal gait, visual disturbances, ascites, bilateral pedal edema, and weakness of bilateral lower limbs. On examination, his Glasgow Coma Scale was 15/15 and was oriented to time, place, and person. Neurological examination revealed the power of 4/5 in all limbs and decreased tone in lower limbs. Laboratory examination revealed polycythemia. Family and personal history of any genetic diseases were unremarkable. There was no history of any chronic diseases and substance abuse. Differentials like cerebrovascular accident, cerebellar dysfunction and substance abuse were ruled out. The rest of the systemic exanimation was normal. MRI brain and spine showed cerebellar hemangioblastoma (Fig. 1) and C2/C3/C4 cervical spinal hemangioblastoma (Fig. 2) respectively. Fundoscopy revealed left retinal angioma. Contrast-enhanced CT abdomen disclosed a malignant space-occupying lesion in segment VI of the liver (Fig. 3) and bilateral renal cortical cysts. Biopsy of the liver mass confirmed capillary liver hemangioblastoma. Genetic testing was not done due to its unavailability. With the aforementioned clinical and radiological findings, a diagnosis of Von Hippel- Lindau disease was established. Midline suboccipital craniectomy was done and brain hemangioblastoma was removed by transfollial approach. Spinal hemangioblastoma embolisation was done. Liver mass was resected in two different settings. Retinal angioma and cortical cysts are regularly followed-up. If the retinal lesion increases, laser photocoagulation is planned. At a 3-month follow-up in outpatient setting, he improved drastically with a marked alleviation of his signs and symptoms.

## 3 Discussion

VHL is characterized by the creation of various benign and malignant tumors, as well as cysts in multiple organs, and is passed down through generations in an autosomal dominant pattern with near-complete penetrance. Currently, clinical diagnostic criteria include the following manifestations: CNS hemangioblastoma (including retinal hemangioblastoma), ELST, RCC, pheochromocytoma, paraganglioma, and neuroendocrine neoplasm [8].

VHL is diagnosed when an individual exhibits the following set of symptoms of genetic/family factors:

• at least two CNS hemangioblastomas
• at least one CNS hemangioblastoma and one other manifestation described above
• at least one of the manifestations described above, and a pathogenic mutation in the *VHL* gene or a first-degree relative with VHL.

A VHL mutation may now be discovered in about 100 % of patients using breakthroughs in genetic testing, such as DNA sequencing and semiquantitative Southern blotting [9]. Because of the unavailability of genetic testing in our setup, it was not done.

Hemangioblastomas of the CNS are one of the most common manifestations of VHL disease. Up to 72 % of VHL patients may have a CNS hemangioblastoma in the cerebellum (16–69 %), brainstem (5–22 %), spinal cord (13–53 %), cauda equina (11 %), or supratentorial (1–7 %) region [10]. Patients with these lesions often present in their second or third decade. CNS hemangioblastomas are the most prevalent (30–35 %) and are frequently the initial symptom of VHL [8]. Despite their benign nature, hemangioblastomas are a substantial cause of morbidity and death in VHL patients because of their mass effect on CNS structures [8]. The gold standard for identifying and monitoring CNS hemangioblastomas is contrast-enhanced magnetic resonance imaging [11]. Microsurgical resection is the preferred therapy for CNS hemangioblastomas. In most circumstances, hemangioblastomas may be safely and completely removed [12]. Preoperative embolization to minimize intraoperative bleeding is not often performed and may pose extra risks [13]. Our patient had cerebellar and spinal hemangioblastomas. Midline suboccipital craniectomy was done and brain hemangioblastoma was removed by transfollial approach. Spinal hemangioblastoma embolization was done.

Retinal hemangioblastomas are quite common in VHL patients. VHL patients will develop retinal hemangioblastomas in between 49 %–62 % of cases [8]. The cornerstones of surgical care for retinal hemangioblastomas are laser photocoagulation and cryotherapy. Because the retinal angioma was small in size, he is regularly followed up. If it increases, laser photocoagulation is planned.

RCC or renal cysts are only seldom (less than 7 % of the time) the first symptom of VHL. Large RCCs may show with traditional indications of a renal mass, such as flank discomfort, hematuria, and/or a flank mass, when present. Simple renal cysts are usually asymptomatic; however complicated cysts can develop into solid RCC tumors. Despite many renal cysts, VHL individuals may preserve renal function [8]. The renal cortical cysts were of simple type in our case. Hence, no surgical intervention was needed. Therefore, they are regularly followed up.

Prenatal and preimplantation genetic diagnostics should be offered for at-risk pregnancies (either parent with known VHL illness or mutation) [14]. Referral to a genetic counselor is suggested to avoid misunderstanding of test findings. Routine observation and early identification help to reduce the secondary consequences of VHL symptoms. Tobacco use and other pollutants may raise the chance of developing kidney cancer. Contact sports should be avoided if you have adrenal or pancreatic lesions [15].

## 4 Conclusions

VHL disease is an extremely complicated disease with the need for diagnosis and genetic tests in the patient and family members, as well as intensive supervision of carriers of the mutated gene, thereby improving early diagnosis and successful treatment of the malignancies. The high cost of diagnostics and surgical therapies is a severe issue. Patients with VHL syndrome should be cared for by well-trained experts and at genetic facilities, and they require psychological help from psychologists and familial support groups. Government care and financial assistance are critical considerations.

## Declaration of competing interest

None.
